# User Engagement Associated with Web-Intervention Features to Attain Clinically Meaningful Weight Loss and Weight Maintenance in Rural Women

**DOI:** 10.1155/2019/7932750

**Published:** 2019-03-03

**Authors:** Patricia A. Hageman, Joseph E. Mroz, Michael A. Yoerger, Carol H. Pullen

**Affiliations:** ^1^Physical Therapy Education, College of Allied Health Professions, University of Nebraska Medical Center, 984420 Nebraska Medical Center, Omaha, NE 68198-4420, USA; ^2^College of Nursing, University of Nebraska Medical Center, 985330 Nebraska Medical Center, Omaha, NE 68198-5330, USA

## Abstract

**Objective:**

Purely web-based weight loss and weight-loss maintenance interventions show promise to influence behavior change. Yet, little is known about user engagement with features of web-based interventions that predict clinically meaningful weight loss (≥5% bodyweight loss). This study examines level of website feature engagement with the likelihood of attaining ≥5% bodyweight loss after 6 and 18 months participation in a web-based intervention, among rural women at high risk of obesity-related diseases and disability.

**Methods:**

In this secondary analysis of clinical trial data of 201 rural women, we examined weight change and user engagement, measured as clicks on specific web-based intervention features (messaging and self-tracking), as associated with clinically meaningful weight loss (baseline to 6 months) and weight-loss maintenance (6 to 18 months).

**Results:**

Generalized estimating equations, adjusted for age, intervention group, and intervention phase, revealed high engagement with messaging predicted whether women achieved ≥5% weight loss at 6 months and at 18 months. There was no effect of self-tracking.

**Conclusions:**

Being engaged with messages was associated with attaining clinically meaningful short-term and longer-term weight loss. This trial is registered with NCT01307644.

## 1. Introduction

Web-based weight loss and weight maintenance interventions are increasingly used to reach individuals who may lack access to programs, such as women from rural populations, as these interventions offer flexibility and scalability to address the public health issue of obesity [1–3]. Purely web-delivered interventions are effective at achieving weight loss [1, 4]; noting the level of participant engagement with web interventions is positively associated with weight-loss success [1, 4–6]. In order to optimize the usage and efficacy of online weight-loss programs, it is important to identify web features that promote clinically meaningful weight-loss outcomes (≥5% bodyweight loss) [6–9].

Emerging evidence suggests that specific web features, such as theory-based behavior-change messaging, and self-monitoring may enhance user engagement and intervention adherence [4, 8, 10]. A major challenge with web-based weight interventions is sustaining sufficient participant engagement over an extended period to facilitate weight loss maintenance, as weight regain often occurs in the months following initial weight loss [11, 12]. One of the gaps in existing literature is the lack of assessment of engagement with specific web features and especially as compared to one another, associated with attaining clinically meaningful short-term weight-loss and longer-term weight-loss maintenance necessary for achieving health outcomes [4, 7].

In our community-based trial that compared the effectiveness of three web-based interventions in rural women, we found women's weight-loss success was correlated to their overall web dosage with the project's basic website that was available to all groups [2]. The proxy for dosage was defined as the count of women's logins by the week of new content delivery, regardless of whether women accessed one or more of the web features. Web dosage was considered an indicator of intervention adherence, defined as usage or exposure to the program as prescribed [5, 13, 14]. The pattern of women's weight loss and web participation was similar across the three groups, with the greatest weight loss and corresponding web dosage observed among 6-month completers. At 18 months, the completers gradually regained weight and web utilization declined by approximately half. As our prior work focused on adherence to the required dosage, it did not take into account women's engagement with specific website features nor did it focus on the level of web engagement necessary for women to achieve clinically meaningful weight-loss targets, which is the intent of this secondary analysis of clinical trial data. Engagement refers to behavioral actions for web usage as related to frequency or duration [13], and for this study, engagement was operationally defined as the frequency or number of “clicks” on a web feature, over two phases of the intervention.

For this analysis, we examined women's level of engagement with intervention website messages and self-tracking features, as associated with women's likelihood of attaining clinically meaningful short-term initial weight loss (baseline to 6 months) and longer-term weight loss maintenance (baseline to 18 months). As self-monitoring such as self-tracking of weight, eating, and activity has been linked to successful weight loss and weight maintenance, we hypothesized that high engagement with this feature would enhance the likelihood of women achieving ≥5% bodyweight loss at 6 and 18 months [15–17]. We anticipated that high engagement with messages would also enhance the likelihood of attaining weight loss targets at 6 and 18 months. As such, we expected the combination of high user engagement with self-tracking and messages would increase the likelihood of weight loss success at each time point.

## 2. Methods

We examined web engagement data, measured as the number of “clicks” on messages and self-tracking features, and weight change over time for this secondary analysis of clinical trial data, from rural women, aged 40–69, enrolled in the Women Weigh-in for Wellness community-based trial. In brief, the primary purpose of the clinical trial was to compare the effectiveness of a web-based intervention alone or supplemented with either peer-led discussion board or professional email counselling, focusing on weight loss (phase 1), guided weight maintenance (phase 2), and self-directed weight maintenance and follow-up (phase 3). The main effect analyses found no group differences in weight loss across time points [2]. We obtained informed consent from all participants, to satisfy all legal and ethical requirements associated with human subject research. The randomized clinical trial from which the data were used for this secondary analysis is registered on https://clinicaltrials.gov/, trial identifier: NCT01307644. Details of the protocol and main effects are published elsewhere [2, 18].

For this secondary analysis, we examined women's level of engagement with web features using data from phase 1 and phase 2 of the intervention, as phase 3 of the trial focused on self-directed weight maintenance and use of the website was optional. Our analyses included women's engagement data from two of the three intervention groups of the parent trial, using women's data from the web-based only group and the group receiving web-based intervention supplemented with email counselling. Women's data from the group receiving web-based intervention supplemented with a peer-led discussion board are not included in our analyses because the information technology analytics for that group were set to tally both “clicks” on the discussion board posts combined with the self-tracking “clicks.” Due to this trial design error for tracking user analytic information, we were unable to separate the women's engagement with self-tracking from their engagement with the website discussion board. Thus, our available sample for the secondary analysis was 201 women out of the 301 trial participants.

### 2.1. Participants

This analysis examined baseline data of 201 women, ages 40–69, who were residents in rural areas of northeastern Nebraska. Of the 201 women, data were available from 179 completers at 6 months and 158 completers at 18 months after baseline assessment. Enrolment and entry of the participants into the study occurred gradually over one year. Eligibility criteria included women having a body mass index of 28–45 kg/m^2^ at baseline and willing to drive up to 70 miles each way to a centrally located research office for required assessments. Women were included if they had web access and confirmed comfort in using email and the Internet. Women were excluded if they were taking medications that affected weight loss or weight gain or if they had a diagnosis of type 1 diabetes or having a diagnosis of type 2 diabetes that required insulin. Other major exclusion criteria included being currently enrolled in a weight loss program, having 10% or greater weight loss in the last 6 months or having any physical or medical restrictions that would preclude following the recommendations for moderate physical activity and healthy eating.

### 2.2. Procedures

Trained research nurses conducted all assessments (baseline, 6 months, and 18 months). All women attended one visit for each assessment period, except at baseline, which required two visits scheduled within a three-week period. At completion of the first baseline visit, the nurses provided each woman with a sealed envelope that contained an identification number and password for the project's website. Between the first and second baseline visits, women were asked to participate in a “practice period” to login and become familiar with the web-site self-tracking features for logging their current weight, food intake, and activity. The web technical support team noted if a woman did not access or use the website during this practice period, and those nonparticipating women were dropped from the study prior to randomization.

### 2.3. Interventions

All women, regardless of intervention group assignment, received access to the same content on the project's website, that included messages and self-tracking features, available during two distinct intervention phases targeting initial weight loss (phase 1) or continued weight loss and weight maintenance (phase 2). Women had access to current and previous messages allowing them the opportunity to revisit items of interest, and they could modify their current and prior self-tracking throughout the intervention. During phase 1 (baseline to 6 months), women received 26 weeks of new content, with an average of 3.6 new messages weekly (range = 1 to 5 weekly messages) for a maximum of 95 messages available. Content focused on healthy eating and activity lifestyle modification to target ≥5% initial bodyweight loss. The lifestyle plan was based upon the 2010 Dietary Guidelines for Americans [19], Healthy People 2010 Recommendations [20], and the 2008 Physical Activity Guidelines for Americans [21]. The messages also focused on benefits and overcoming barriers to action, building self-efficacy for action, finding social support, and goal setting, all of which are constructs of Pender's Health Promotion Model [22]. A complete listing of phase 1 message content is available in the supplemental materials (Supplemental Table 1).

Phase 2 (6 months to 18 months) focused on continued weight loss and weight-loss maintenance for a target bodyweight loss from baseline of ≥5%. In an attempt to keep the women engaged throughout phase 2, a different form of messages was available, called “hot topics.” These messages were based on contemporary stories about current research findings, reported in various social media at the time. One new “hot topic” was available biweekly during 6 to 12 months, and monthly during 12 to 18 months, for a total of 26 weeks of new content. As enrolment of women into the study occurred over one year, at any given time, there were women at differing stages of the intervention, making it likely that women were exposed to a differing array of hot topic messages during phase 2. A complete listing of phase 2 message content is available in the supplemental materials (Supplemental Table 2).

During both phases, women had access to a self-tracking tool, which permitted daily self-tracking of weight, calorie intake, fat grams, pedometer steps, and minutes spent in moderate or greater physical activity, and allowed weekly self-tracking of goals. Women's entries were displayed graphically, so women could visualize their performance over time.

### 2.4. Outcomes

Women provided general demographic information via survey. Weight (kg), height (m), and body mass index (BMI) (kg/m^2^) were assessed using the Tanita scale (TBF-215, Tanita Corporation of American, Inc., Illinois) following the manufacturer's protocol. Percentage weight change was calculated for baseline to 6 months, and baseline to 18 months. To analyse clinically meaningful weight loss, we then coded the percentage of initial weight loss at 6 and 18 months as ≥5% (0 = target not achieved, 1 = target achieved). Website engagement was measured based upon the number of “clicks” on a given web feature, classified as messages or self-tracking, during phase 1 and phase 2 of the intervention. The measure for self-tracking engagement was a combination of the use of the tools for tracking a variety of weight, eating, and activity behaviors and goals.

### 2.5. Analysis

Data from all women, regardless of group assignment in the original trial, were pooled for analysis, as all had access to the same website features. Descriptive statistics were used to report characteristics of the women and their website engagement with messages and self-tracking.

We conducted repeated-measures unweighted logistic regressions using generalized estimating equations (GEEs) in SPSS v25 to compare clinically meaningful weight loss (≥5% of initial weight) based on the degree of engagement (low, moderate, or high) with messaging and self-tracking behaviors. GEEs were used to account for nonindependence of data because women provided data at multiple time points. That is, we had two observations for each woman on weight and engagement, and, therefore, those data are necessarily nonindependent. GEEs also allowed us to use all available data in our analyses, meaning that women who had phase 1 or phase 2 data were included. We estimated three equations—each of which included age, intervention group, and phase as covariates—to separately test the effects of engagement with messages (equation 1), engagement with self-tracking (equation 2), and the combination of messages and self-tracking (equation 3). Age and intervention group were included as covariates because age and web interventions with supplemental features have been associated with web engagement [7, 9, 23]. All equations used robust standard errors, an unstructured correlation matrix, and the kernel of the log quasi-likelihood function to estimate equation fit.

Similar to the work of others [9], the women's engagement data were split into tertiles, such that a third of the sample was classified into a low, moderate, or high group for each facet of engagement (messaging and self-tracking) during phase 1 and phase 2. Given the frequency distribution of the raw variables, the number of women in each tertile of engagement was not always equivalent, as groups of several women often clustered at or near the cut points. We used ANOVAs to test whether the engagement tertile groups differed from one another in terms or age, baseline body mass index, and baseline weight (in kg).

## 3. Results

At baseline, the mean (SD) age was 54.4 (6.9) years for the 201 women whose weight and engagement data were used for this analysis. These women had a baseline mean (SD) BMI of 34.7 (4.2) k/m^2^. The women were primarily Caucasian (98.5%; *n* = 198), employed either full- or part-time (85%; *n* = 171), and had completed some college or higher (82.6%; *n* = 166). The majority were of high socioeconomic status (77.1%; *n* = 155), reporting a household income of >$40,000.

There were 179 completers at 6 months and 158 completers at 18 months. At both 6 and 18 months, the overall percentages of completers for representation of race, employment, education, and household income were similar to those at baseline. At 6 months, the 179 completers lost an average (SD) of 6.1% (6.7%) of their initial weight. Approximately 48% (*n* = 85) of women achieved clinically significant weight loss of ≥5%. Phase 2 (6 to 18 months) focused on continued weight loss and weight maintenance, with 158 women completers at 18 months having an average (SD) of 4.7% (7.8%) of weight loss from baseline (median = 3.1%). Nearly 37% (*n* = 59) of 18-month completers had ≥5% of initial bodyweight loss.

Table 1 includes descriptive statistics of the number of participants assigned to each engagement tertile group, the median raw engagement score for members within the group, and the range/cutoff score that defined group membership. Women in the high engagement group had engagement counts (or clicks) that most closely corresponded with the maximum number of new messages available in phases 1 and 2. By way of example during phase 1, 95 different messages were available, and women with high engagement had a median of 122 message counts, with a range from 99 to 199. Similarly, during phase 2, 26 different hot topic messages were available and women with high engagement had a median of 33 counts, with a range of 19–71 counts.

We also compared engagement groups in phase 1 (baseline to 6 months) to determine equivalence on baseline characteristics. Women who were highly engaged with theory-based behavior-change messages were older (*p* < 0.001) with mean (SD) of 56.5 (6.7) years than those with low engagement 53.3 (6.3) years. Women did not differ with respect to BMI or weight between the messaging engagement tertile groups. Phase 1 self-tracking engagement groups did not differ by age, BMI, or weight at baseline.

### 3.1. Effects of Messaging and Self-Tracking Engagement

Three GEEs were computed, and complete results of each analysis are included in Table 2. Analyses included 180 women: 179 women who had data for phase 1 weight loss, 158 for phase 2 weight loss, and 1 woman who was missing data for phase 1 but whose phase 2 weight was recorded. The first equation tested the effect of messaging on achieving the weight-loss targets while accounting for age, intervention group, and intervention phase. There was no effect of age, and the effect of phase was significant in that women were 1.58 times as likely to achieve the weight-loss target in phase 1 when compared to phase 2 (*b* = 0.44, se = 0.17, *p*=0.008). Women in the web-based with email counselling (web + email) intervention group were 1.79 times more likely to meet the weight-loss targets when compared to the web-only group (*b* = 0.29, se = 0.14, *p*=0.036). Level of engagement with messages was a significant predictor (*p* < 0.001), and results showed that women with low (*b* = −1.37, se = 0.32, *p* < 0.001) and moderate (*b* = −0.97, se = 0.31, *p*=0.002) message engagements were 0.25 and 0.38 times as likely, respectively, to achieve the weight-loss targets when compared to women with high engagement.

The second equation was the same as equation 1 regarding covariates, but it estimated the effect of engagement with self-tracking rather than the effect of engagement with messaging. There was not a significant effect of age or intervention group. Again, women were 1.64 times more likely to meet the weight-loss target in phase 1 than in phase 2 (*b* = 0.49, se = 0.16, *p*=0.002). Engaging with self-tracking was a significant predictor (*p* < 0.001), and findings indicated that women with low engagement were 0.31 times as likely to meet the weight loss targets as highly engaged women (*b* = −1.12, se = 0.29, *p* < 0.001). There was no difference between moderately and highly engaged women.

The last equation estimated the effects of messaging and self-tracking simultaneously, after accounting for age, intervention group, and phase. Age did not exhibit an effect on weight loss, but the intervention group and phase did. Women were 1.65 times as likely to meet the weight-loss target in phase 1 compared to phase 2 (*b* = 0.50, se = 0.16, *p*=0.002). Additionally, women in the web + email intervention group were 1.72 times more likely to achieve weight-loss targets as compared to the web-only group (*b* = 0.27, se = 0.14, *p*=0.046). There was no unique effect of self-tracking, but message engagement remained significant (*p* < 0.001) with highly engaged women being 3.75 and 2.33 times more likely to meet weight-loss targets when compared to women with low (*b* = −1.32, se = 0.34, *p* < 0.001) or moderate (*b* = −0.85, se = 0.31, *p*=0.007) engagement.

## 4. Discussion

This is one of the few papers found that assesses the association between the level of user engagement with specific features of a purely web-delivered intervention with attaining clinically meaningful short-term or longer-term weight loss of ≥5%. When considered separately in a repeated-measures analysis and accounting for age, intervention group, and intervention phase, our findings show weight loss of ≥5% at 6 months and at 18 months was associated with greater engagement with all website features at phases 1 or 2. However, when engagement with both messaging and self-tracking was included in the analysis simultaneously, only high engagement with messaging was associated with attaining ≥5% weight loss.

Consistent with findings from other studies [9, 23, 24], our women who had high engagement with messages were older and highly educated; however, we did not find an effect of age on whether women met the weight-loss target of ≥5%. Perhaps, the messages may have been more meaningful or appealing for older women, or perhaps these women felt a strong sense of duty to view messages.

Typically, long-term engagement with any weight maintenance program is challenging and may be especially so in purely web-delivered programs where the novelty of the website is waning, or when the content provided is not always suitable to meet the users' needs [25, 26]. New messages were posted frequently, but there were limited updates to the overall layout of the intervention home page. Our retention of women between 6 and 18 months was relatively high (89% and 79%, respectively), and it is possible that the change in content and focus of our messages between the phases helped in our retention of women.

Based on prior research, we expected engagement with self-tracking would be a powerful predictor of weight loss [8, 15–17]. Self-tracking was a significant predictor of achieving the weight loss outcome when it was the only predictor in the model aside from the covariates. Contrary to our expectation, self-tracking was not associated with ≥5% weight loss in a model that also considered engagement with messaging. This finding suggests that, at least in our study, women who engaged highly with messages were likely to meet the weight-loss target regardless of how much self-tracking they engaged in. One reason for this finding may be that women who were highly engaged with messages were also highly engaged with self-tracking such that it was statistically difficult to find a unique effect of tracking.

It remains unclear whether moderate or low engagement with the web features influenced women's outcomes or perhaps, the women's awareness of being in a clinical study or other factors influenced their behaviors for weight loss success. The literature suggests that small, nonclinically significant weight losses achieved during participation in web interventions may have benefits on the public health level [3]. While the web offers a promising avenue for delivering behavior-change interventions, there is lack of knowledge about how to maintain web engagement, and there is a paucity of literature in understanding the relationship between web engagement and achieving the desired intervention health outcome [13, 14, 24, 27].

A strength of this study is longer-term retention (18 months) of a large sample of hard-to-reach rural women, who are at high risk for obesity-related diseases and disability. The web intervention included features shown to have success, such as theory-based messaging and self-monitoring through visual representation of tracking weight, eating, and activity. Engagement with features of web-based interventions may enhance the participant's sense of control and potentially reduce dropouts [15–17]. Another strength of this study is that it considered the effects of messaging and self-tracking simultaneously. This controlled for shared variance between the two engagement factors, thereby answering the question of whether engagement with both features is a significant predictor or whether one is superior to the other. Whereas the majority of purely web-based interventions are focused on short-term weight loss [1, 3, 4]; this study also included a weight maintenance intervention. This study is one of only a few found that examined user engagement with different web features associated with attaining clinically meaningful weight-loss targets [4].

The primary limitation of this study was the ability to discern user engagement, as the use of “clicks” as a proxy for engagement may not accurately capture whether women read the messages. We did not have the capability to assess whether women viewed new messages or revisited selected messages. We lacked the ability to differentiate which specific self-tracking feature was used, whether weight, eating, activity, or goal setting. Our discovery of potential miscounting of “clicks” for the self-tracking subgroup of women receiving the web plus peer-led discussion board intervention is a limitation; however, the remaining sample size was notable for 6- and 18-month analyses. While retention of women in the study was relatively high compared to other studies, potential reasons for women dropping from the study might be lack of interest in the website, limited motivation to follow the recommended interventions, or limited Internet access. Similar to other web-based interventions, the sample of women were Caucasian and of higher socioeconomic status, limiting the generalizability of the results [1, 3, 24].

## 5. Conclusions

As researchers have highlighted the need to examine which specific elements of web-based interventions enhance the impact on outcome measures, this analysis found that women with high engagement with messaging were most likely to achieve clinically significant weight loss and weight maintenance targets of ≥5% at 6 months or at 18 months. Although engagement with messaging and self-tracking predicted weight loss when examined separately, combining both into the same model revealed that only engagement with messaging exhibited a unique effect. Future research appears warranted to identify web intervention features essential to achieve efficacious weight loss and weight maintenance, including a determination of optimal user engagement to achieve this goal. As technology capabilities are rapidly evolving, there is a need for more in-depth and standardized assessments about web-user behaviors and their influences.

## Figures and Tables

**Table 1:** Women's level of web-feature engagement^a^ (tertiles) by intervention phase.

Web feature	Low		Moderate		High	
	Median (*n*)	Range	Median (*n*)	Range	Median (*n*)	Range
*Phase 1* ^b^ initial weight loss (baseline–6 months)						
Messages	21.5 (60)	0–49	79.5 (62)	51–98	122 (57)	99–199
Self-tracking	25 (60)	1–43	64 (62)	44–77	103 (57)	78–230
*Phase 2* ^c^ weight maintenance (6–18 months)						
Hot topic messages	0 (55)	0–2	9 (43)	3–18	33 (61)	19–71
Self-tracking	4 (49)	0–14	47 (51)	15–60	78 (59)	61–144

^a^Engagement was measured by the number of “clicks” on a web-feature. Median is displayed due to nonnormal distributions and (*n*) represents the number of individuals in a tertile, noting tertile numbers may vary due to frequency of “clicks” clustered at or near the cut points. ^b^Phase 1: women received a range of 1–5 new theory-based messages per week, posted once weekly over 26 weeks, for a total of 95. ^c^Phase 2: women received 26 new hot topic messages posted biweekly during months 6 to 12 and posted monthly from months 12 to 18.

**Table 2:** Effects of age, intervention group, time (phase), message engagement, and self-tracking engagement on achieving ≥5% weight loss.

Equation	Parameter estimate (95% confidence interval)	Standard error	Wald chi-square
*Equation 1: messaging*			
Age	0.01 (−2.67, 1.69)	0.02	0.37
Web-only vs web + email intervention	0.29 (0.02, 0.56)	0.14	4.41^*∗*^
Phase	0.44 (0.12, 0.77)	0.17	7.08^*∗∗*^
Low vs high message engagement	−1.37 (−1.99, −0.75)	0.32	18.80^*∗∗∗*^
Moderate vs high message engagement	−0.97 (−1.58, −0.37)	0.31	10.00^*∗*^
*QIC fit estimate: 432.67*			
*Equation 2: tracking*			
Age	0.03 (−0.01, 0.06)	0.02	1.55
Web-only vs web + email intervention	0.25 (−0.02, 0.53)	0.14	3.40
Phase	0.49 (0.18, 0.80)	0.16	9.76^*∗∗*^
Low vs high tracking engagement	−1.12 (−1.68, −0.55)	0.29	15.18^*∗∗∗*^
Moderate vs high tracking engagement	−0.42 (−0.94, 0.09)	0.26	2.57
*QIC fit estimate: 443.32*			
*Equation 3: messaging and tracking*			
Age	0.01 (−0.03, 0.05)	0.02	0.09
Web-only vs web + email intervention	0.27 (0.01, 0.54)	0.14	3.98^*∗*^
Phase	0.50 (0.19, 0.82)	0.16	9.92^*∗∗*^
Low vs high message engagement	−1.32 (−1.98, −0.67)	0.34	15.55^*∗∗∗*^
Moderate vs high message engagement	−0.85 (−1.46, −0.24)	0.31	7.35^*∗∗*^
Low vs high tracking engagement	−0.50 (−1.11, 0.11)	0.31	2.60
Moderate vs high tracking engagement	−0.08 (−0.64, 0.48)	0.28	0.08
*QIC fit estimate: 431.67*			

*Note.* 180 women used in analyses. QIC = quasi-likelihood under independence model criterion. For phase, phase 1 (baseline to 6 months) = 0 and phase 2 (6 months to 18 months) = 1. ^*∗*^ *p* < 0.05. ^*∗∗*^ *p* < 0.01. ^*∗∗∗*^ *p* < 0.001.

## Data Availability

The data, available in SPSS v25, used to support the findings of this study are available from the corresponding author upon reasonable request.

## Supplementary Materials

Supplementary Materials Supplemental Materials include web-based messaging content delivered during two phases of the intervention. Supplementary Table 1: website theory-based message intervention content for phase 1 (baseline to 6 months). Supplementary Table 2: website “Hot Topic” message intervention content for phase 2 (6 months to 18 months).

## References

[1] Sorgente A., Pietrabissa G., Manzoni G. M. (2017). Web-based interventions for weight loss or weight loss maintenance in overweight or obese people: a systematic review of systematic reviews. *Journal of Medical Internet Research*.

[2] Hageman P. A., Pullen C. H., Hertzog M., Pozehl B., Eisenhauer C., S Boeckner L. (2017). Web-based interventions alone or supplemented with peer-led support or professional email counseling for weight loss and weight maintenance in women from rural communities: results of a clinical trial. *Journal of Obesity*.

[3] Wieland L. S., Falzon L., Sciamanna C. N. (2012). Interactive computer-based interventions for weight loss or weight maintenance in overweight or obese people. *Cochrane Database of Systematic Reviews*.

[4] Neve M., Morgan P. J., Jones P. R., Collins C. E. (2010). Effectiveness of web-based interventions in achieving weight loss and weight loss maintenance in overweight and obese adults: a systematic review with meta-analysis. *Obesity Reviews*.

[5] Cussler E. C., Teixeira P. J., Going S. B. (2008). Maintenance of weight loss in overweight middle-aged women through the internet. *Obesity*.

[6] Hwang K. O., Ning J., Trickey A. W., Sciamanna C. N. (2013). Website usage and weight loss in a free commercial online weight loss program: retrospective cohort study. *Journal of Medical Internet Research*.

[7] Collins C. E., Morgan P. J., Hutchesson M. J., Callister R. (2013). Efficacy of standard versus enhanced features in a web-based commercial weight-loss program for obese adults, part 2: randomized controlled trial. *Journal of Medical Internet Research*.

[8] Krukowski R. A., Harvey-Berino J., Ashikaga T., Thomas C. S., Micco N. (2008). Internet-based weight control: the relationship between web features and weight loss. *Telemedicine and e-Health*.

[9] Johnson F., Wardle J. (2011). The association between weight loss and engagement with a web-based food and exercise diary in a commercial weight loss programme: a retrospective analysis. *International Journal of Behavioral Nutrition and Physical Activity*.

[10] Khaylis A., Yiaslas T., Bergstrom J., Gore-Felton C. (2010). A review of efficacious technology-based weight-loss interventions: five key components. *Telemedicine and e-Health*.

[11] MacLean P. S., Wing R. R., Davidson T. (2015). NIH working group report: innovative research to improve maintenance of weight loss. *Obesity*.

[12] Pekkarinen T., Kaukua J., Mustajoki P. (2015). Long-term weight maintenance after a 17-week weight loss intervention with or without a one-year maintenance program: a randomized controlled trial. *Journal of Obesity*.

[13] Ryan C., Bergin M., Wells J. S. (2017). Theoretical perspectives of adherence to web-based interventions: a scoping review. *International Journal of Behavioral Medicine*.

[14] Beatty L., Binnion C. (2016). A systematic review of predictors of, and reasons for, adherence to online psychological interventions. *International Journal of Behavioral Medicine*.

[15] Levine D. M., Savarimuthu S., Squires A., Nicholson J., Jay M. (2015). Technology-assisted weight loss interventions in primary care: a systematic review. *Journal of General Internal Medicine*.

[16] Manzoni G. M., Pagnini F., Corti S., Molinari E., Castelnuovo G. (2011). Internet-based behavioral interventions for obesity: an updated systematic review. *Clinical Practice in Epidemology Mental Health*.

[17] Raaijmakers L. C. H., Pouwels S., Berghuis K. A., Nienhuijs S. W. (2015). Technology-based interventions in the treatment of overweight and obesity: a systematic review. *Appetite*.

[18] Hageman P. A., Pullen C. H., Hertzog M., Boeckner L. S., Walker S. N. (2011). Web-based interventions for weight loss and weight maintenance among rural midlife and older women: protocol for a randomized controlled trial. *BMC Public Health*.

[19] (2010). *Dietary Guidelines for Americans*.

[20] (2010). Healthy people 2010: with understanding and improving health.

[21] (2008). *Physical Activity Guidelines for Americans*.

[22] Pender N., Murdaugh C., Parsons M. (2006). *Health Promotion in Nursing Practice*.

[23] Brindal E., Freyne J., Saunders I., Berkovsky S., Smith G., Noakes M. (2012). Features predicting weight loss in overweight or obese participants in a web-based intervention: randomized trial. *Journal of Medical Internet Research*.

[24] Kelders S. M., Van Gemert-Pijnen J. E. W. C., Werkman A., Nijland N., Seydel E. R. (2011). Effectiveness of a web-based intervention aimed at healthy dietary and physical activity behavior: a randomized controlled trial about users and usage. *Journal of Medical Internet Research*.

[25] Arem H., Irwin M. (2011). A review of web-based weight loss interventions in adults. *Obesity Reviews*.

[26] Eysenbach G. (2005). The law of attrition. *Journal of Medical Internet Research*.

[27] Davies C., Corry K., Van Itallie A., Vandelanotte C., Caperchione C., Mummery W. K. (2012). Prospective associations between intervention components and website engagement in a publicly available physical activity website: the case of 10,000 steps Australia. *Journal of Medical Internet Research*.

