# Supplementary material for: User Engagement Associated with Web-Intervention Features to Attain Clinically Meaningful Weight Loss and Weight Maintenance in Rural Women
Source: J Obes. 2019 Mar 3;2019:7932750. doi: 10.1155/2019/7932750 (PMC6421718; doi:10.1155/2019/7932750)
Supplement: Supplementary Materials — Supplemental Materials include web-based messaging content delivered during two phases of the intervention. Supplementary Table 1: website theory-based message intervention content for phase 1 (baseline to 6 months). Supplementary Table 2: website “Hot Topic” message intervention content for phase 2 (6 months to 18 months). [file 7932750.f1.pdf]

Journal of Obesity
Manuscript 7932750

Supplemental Materials: Materials include web-based messaging content delivered during two phases of the intervention.

Supplementary Table 1: Website Theory-Based Message Intervention Content for Phase 1 (baseline to 6 months).

Supplementary Table 2: Website "Hot Topic" Message Intervention Content for Phase 2 (6 months to 18 months).

Table 1.Website Theory-Based Message Intervention Content-Phase 1 (baseline to 6 months)
Women Weigh-In for Wellness Study (WWW Study)

In Phase 1 (Guided Weight Loss), women in all groups (WO, WD, & WE) had access to the same content on the trial website, representing the identified constructs as noted. Women received automated email messages when new web content was available, scheduled weekly during Phase 1.

Week	Content	Info			Benefits Barriers		Self Efficacy			IP Support		Self Monitor	Goals
		G	PA	Eat	PA	Eat	PA	Eat	O	PA	Eat		
	Tips for Navigating on the Site	X										X	X
	6S's (Success) - Self commit, Self Monitor, SMART GOAL, Eat, Start Moving, and Social support		X	X								X	X
	WWW Plan for YOU	X	X									X	
	Weighing Yourself - How Hard Can It Be?	X										X	
0	Getting Through Your Practice Week	X											
	Ready? Set? Let's Go!											X	X
	Your Weight Loss Plan Revealed	X	X	X									
	Easing Into Starting to Move		X		X		X						
	Shop to Drop (Pounds That Is)			X				X					
1	Gentle Reminder - Keep Group Activities Confidential	X											
	Congratulations - You Survived Your First Week	X							X			X	X
	Making the Right Moves for Weight Loss		X				X						
	Portion Distortion Quiz (NHLBI)			X				X					
	Raise Your Arms - Goals!												X
2	Lean on Me - Support									X	X		
	A-Ha Moments	X					X	X				X	
	Start Moving - Plan for Physical Activity	X	X		X		X						
	Start Moving Log: Keeping a Daily Tally		X									X	
3	Setting SMART Goals	X	X	X									X
	Mindful Weight Loss				X	X	X	X					
4	True Success Story					X		X					
	Celebration, Tune-Up, or Re-Set	X					X	X	X	X	X	X	X
5	Am I Really Hungry?					X		X					
	PLAN So you can Stick to the Plan						X	X	X			X	X
	Your Plan Can Save the Day!				X	X					X		
6	You Deserve a Reward - Cookbook						X	X	X				
	Let's Really Get Moving!		X		X		X			X			
	Goal Challenge: Go for the Gold!												X
7	Physical Activity Rewards: Peggy's Story				X		X						
	How to Overcome Physical Activity Barriers				X								
	Physical Activity is Enjoyable!				X								
	Writing Physical Activity Goals												X
8	Breakfast Recipe!			X									
	Weight Loss Stalled? - Jump Start Guide	X	X	X								X	
	Portion Distortion Quiz - Part 2			X				X					
9	Bonus Tip to Get off a Plateau	X										X	

Legend: G - General O - Other
PA - Physical Activity IP - Interpersonal

Table 1.Website Theory-Based Message Intervention Content-Phase 1 (baseline to 6 months)
Women Weigh-In for Wellness Study (WWW Study)

Week	Content	Info			Benefits Barriers		Self Efficacy			IP Support		Self Monitor	Goals
		G	PA	Eat	PA	Eat	PA	Eat	O	PA	Eat		
	Sleep & Weight Connection	X											
9	Avoid Food Habits that Interfere with Your Sleep			X		X		X			X		
10	Regular Exercise Helps you Sleep Better		X		X								
	Stress Can Interfere with your Weight Loss	X			X	X							
	Barriers of Stress				X	X				X	X		
	Success Story: Mary Kay's A-Ha Moment									X	X		
11	Need a Healthy Snack?			X									
	Look at Your New Normal Lifestyle	X					X	X	X				
	Finding it difficult to meet your activity goals?				X								
	Limit Screen Time to Lose Weight				X								
12	I Love Vegetables Recipe			X									
	Your New Lifestyle and 6S Behaviors	X					X	X	X			X	X
	Use the 6S Plan to Enjoy Social Gatherings										X		
	Sensible Tips to Eating Out										X		
13	Smarter Eating Tips by Type of Restaurant			X		X		X				X	
	Clean out your closet for success	X							X			X	
	Forget Fatigue - Find Energy		X		X		X			X			
14	Breakfast Fruit Cup Recipe			X									
	Transformation of Mind and Body	X							X				X
	Plan Your Personal Success Story	X											X
	Fine Tune Your Efforts to be More Active for life		X		X		X			X		X	
15	Success Story: Betty's Successful Activity Habit						X	X					
	Get Re-Motivated & Energized	X											
	5 Ways to Beat Bordem with Physical Activity		X		X								
	Be Greatful that you can do physical activity						X						
	Updated Nutrition Guide with Suggested Servings			X				X				X	
16	MyPlate Guide for a Balanced Diet			X									
	Do you LOVE a bargain?	X							X				
	Health Costs of Overweight	X			X	X							
	EAT BREAKFAST!			X									
17	Nutrition Corner as a Rainbow			X		X							
	Super S! Challenging Situations				X	X	X	X	X	X	X		
	No Couch Potato!		X		X		X			X			
18	Nutrition Corner: Fabulous Fruits!			X		X							
	Busting Weight Loss Myths	X					X	X	X				

Legend: G - General O - Other
PA - Physical Activity IP - Interpersonal

Table 1.Website Theory-Based Message Intervention Content-Phase 1 (baseline to 6 months)
Women Weigh-In for Wellness Study (WWW Study)

Week	Content	Info			Benefits Barriers		Self Efficacy			IP Support		Self Monitor	Goals
		G	PA	Eat	PA	Eat	PA	Eat	O	PA	Eat		
	There may be weight loss saboteures									X	X		
	An Active Life Feels GOOD!				X								
	Success Story: Straight Talking Kathy						X	X	X			X	X
19	Nutrition Corner: Dairy Foods, Calcium, & Vitamin D			X		X							
	Staying Power						X	X	X				
	Astounding Challenge: Party AND Stay on Track				X	X						X	X
	10 Minute Activity Challenges		X		X								
	Slow & Steady Wins						X	X					
20	Nutrition Corner: Whole Grains			X		X							
	Moods: What to do with them	X					X	X	X				
	Living Well at Any Age				X	X							
21	Nutrition Corner: Protein			X		X							
	Take an Inventory of You				X	X	X	X		X	X	X	X
	Learning to Love Activity				X		X			X			
22	Not So Sweet Talk About Sugar			X									
	Look in the Mirror: New Appearance and New Thinking	X					X	X	X	X	X		
	Physical Activity: Keep it Interesting				X		X			X			
23	Nutrition Corner: Fat, it isn't all bad			X		X							
	Thank You & About Phase II	X	X	X									
	Where am I?											X	X
24	Be Happy Whatever Situation You Find Yourself In	X							X				
	Phase II - How It Works	X											
	Weight Loss & Weight Maintenance		X	X									
25	Record Your Goals to Be What You Want to Be												X

Legend: G - General; O - Other; PA - Physical Activity; IP - Interpersonal

Table 2.Website "Hot Topic" Message Intervention Content-Phase 2 (6 months to 18 months)
Women Weigh-In for Wellness Study (WWW Study)

For Phase 2 (Guided Weight Loss and Weight Maintenance), women in all groups (WO, WD, WE) had access to the same content on the trial website, representing the identified constructs as noted. Women received automated email messages when new web content was available (biweekly), called “Hot Topics” in reference to their focus on current information or events in the news.

Week	Content	Info			Benefits Barriers		Self Efficacy			IP Support		Self Monitor	Goals
		G	PA	at	PA	Eat	PA	Eat	O	PA	Eat		
27	Want to Cut Chocolate Cravings? Take a Walk[1]	X		X		X					X		
29	Poor Lifestyles Harming U.S. Heart Health: Report[1]				X	X							
31	Family Tips for Getting Fit[1]									X			
33	What Are Coronary Heart Disease Risk Factors?[2]	X			X	X							
35	When Prodded, Restaurant Diners Often Opt for Smaller Portions[1]			X				X				X	
37	Desk Jockeys Urged to Take Small Steps to Get Exercise[1]				X		X					X	
39	Fruits, Veggies Can Be Beauty Tools, Study Says[1]			X		X							
41	Too Much Sitting Can Kill You, Study Suggests[1]		X		X								
	Health Tip: Why You Need Aerobic Exercise[1]		X		X								
43	Could Two Words Help You Resist Temptation?[1]					X		X					
45	Women Less Apt Than Men to Get Recommended Daily Exercise[1]		X		X								
	Health Tip: Overcoming Obstacles to Exercise[1]												
47	Healthy Weight Loss May Also Cut Your Cancer Risk[1]	X			X	X							
	Keeping It Off[3]									X	X	X	
49	Stop the Cravings![4]							X			X	X	
51	Stay Active and Save Your Life![5]		X		X		X			X			
	Go4Life[6] & Success Stories				X	X	X	X	X				
53	National Weight Control Registry Facts & Success Stories[7]				X	X	X	X	X	X	X	X	X
55	GMA Medical Minute & Food Is A Numbers Game[8]								X			X	X
57	Want to Live Longer? Turn Off That TV and Stand Up[1]		X		X							X	
	Guide to Physical Activity[2]		X										

Legend: G - General O - Other
PA - Physical Activity IP - Interpersonal

Table 2.Website "Hot Topic" Message Intervention Content-Phase 2 (6 months to 18 months)
Women Weigh-In for Wellness Study (WWW Study)

59	Shape Up, Safely, for Summer & Health Tip: Exercising in the Heat[1]	X	X		X	X	X	X	X	X		X	
61	Recipies to Support Your New Lifestyle[9]			X		X		X					
63	RedHot News from the American Heart Association: Go Red for Women[10]	X	X	X	X	X	X	X		X	X		
65	Lettuce: Buy Bagged or Not?[1]			X									
		Info			Benefits Barriers		Self Efficacy			IP Support			
**Week**	**Content**	**G**	**PA**	**Eat**	**PA**	**Eat**	**PA**	**Eat**	**O**	**PA**	**Eat**	**Self Monitor**	**Goals**
67	Weight Loss Dos and Don'ts Quiz[9]	X	X	X			X	X					
69	Slideshow: 10 Ways to Boost Your Metabolism[9]	X	X	X	X	X							
71	Fast Walking May Slash at Heart Disease, Diabetes[9]		X		X								
73	Diet/Exercise Combo Best, 'Biggest Loser' Study Says[1]	X	X	X									
75	Says[1]		X		X								
	Holiday Eating Guide[10]			X		X		X		X	X	X	
77	Says[1]		X		X								
79	Stay Active Through the Winter[11]		X		X								
81	Inspiration for a Healthy New Year[3] & Success Stories						X	X	X	X	X		
83	Berries May Cut Heart Attack Risk in Women, Study Says[1]			X		X							
85	Weight Control[5]	X	X	X	X	X							
87	Smaller Snack Portions Just as Satisfying[1]			X				X					
89	Everyday Activities May Have Same Health Benefits as Going to Gym[1]		X		X		X						
91	Weight[9]	X	X									X	
93	Don't Just Sit There![12]		X		X								

References:
1. healthfinder.gov
2. National Heart Lung and Blood Institute
3. Centers for Disease Control and Prevention
4. Academy of Nutrition and Dietetics
5. NIH MedlinePlus
6. National Institute on Aging at NIH
7. The National Weight Control Registry
8. ABC News
9. WebMD
10. American Heart Association
11. Department of Health and Human Services
12. NIH News in Health

Legend: G - General; O - Other; PA - Physical Activity; IP - Interpersonal
